# Mediation of episodic memory performance by the executive function network in patients with amnestic mild cognitive impairment: a resting-state functional MRI study

**DOI:** 10.18632/oncotarget.11775

**Published:** 2016-08-31

**Authors:** Baoyu Yuan, Jiu Chen, Liang Gong, Hao Shu, Wenxiang Liao, Zan Wang, Duan Liu, Chunming Xie, Zhijun Zhang

**Affiliations:** ^1^ Department of Neurology, ZhongDa Hospital, Neuropsychiatric Institute, Medical School of Southeast University, Nanjing, PR China

**Keywords:** amnestic mild cognitive impairment, executive function, episodic memory, regional homogeneity, functional magnetic resonance imaging, Pathology Section

## Abstract

Deficits in episodic memory (EM) are a hallmark clinical symptom of patients with amnestic mild cognitive impairment (aMCI). Impairments in executive function (EF) are widely considered to exacerbate memory deficits and to increase the risk of conversion from aMCI to Alzheimer's disease (AD). However, the specific mechanisms underlying the interaction between executive dysfunction and memory deficits in aMCI patients remain unclear. Thus, the present study utilized resting-state functional magnetic resonance imaging (fMRI) scans of the EF network and the EM network to investigate this relationship in 79 aMCI patients and 119 healthy controls (HC). The seeds were obtained from the results of a regional homogeneity (ReHo) analysis. Functional connectivity (FC) within the EM network was determined using a seed in the right retrosplenial cortex (RSC), and FC within EF network was assessed using seeds in the right dorsolateral prefrontal cortex (DLPFC). There was a significant negative correlation between EM scores and EF scores in both the aMCI and HC groups. Compared to the HC group, aMCI patients had reduced right RSC connectivity but enhanced right DLPFC connectivity. The overlapping brain regions between the EM and EF networks were associated with FC in the right inferior parietal lobule (IPL) in the right RSC network, and in the bilateral middle cingulate cortex (MCC) and left IPL in the right DLPFC network. A mediation analysis revealed that the EF network had an indirect positive effect on EM performance in the aMCI patients. The present findings provide new insights into the neural mechanisms underlying the interaction between impaired EF and memory deficits in aMCI patients and suggest that the EF network may mediate EM performance in this population.

## INTRODUCTION

It has been proposed that amnesic mild cognitive impairment (aMCI) is a condition of intermediate symptomatology that represents the cognitive changes between normal aging and very early dementia in populations at high risk of Alzheimer's disease (AD) [1]. It is well known that deficits in episodic memory (EM) are a hallmark clinical symptom of aMCI [2, 3], and it has been consistently demonstrated that the simultaneous existence of impaired executive function (EF) may also be present in these patients [4–7]. In particular, impaired EF exacerbates memory deficits and may increase the risk of conversion from aMCI to AD [8, 9]. However, little is currently known about the relationship between EF and EM in aMCI patients.

Converging lines of evidence strongly indicate that the deposition of β-amyloid (Aβ) proteins occurs within the default mode network (DMN) [10–12] and that this process is associated with impaired resting-state connectivity [13–15]. It is well established that the DMN is involved with EM [16–18]. Additionally, memory function is not only facilitated by the medial temporal lobe (MTL) system but is supported by a distributed network, particularly in the DMN [19]. Structural and functional imaging studies have consistently established that the retrosplenial cortex (RSC) and the posterior cingulate cortex (PCC) are the main hubs connecting with the MTL and that the prefrontal cortex (PFC) plays a central role in processing EM [20–22].

Numerous studies have observed changes in resting-state functional connectivity (RSFC) within the DMN of patients with aMCI [23–30]. Additionally, in clinical terms, several functional magnetic resonance imaging (fMRI) studies have reported that the altered integrity of the DMN is related to memory impairments and that the PCC and RSC are among the brain regions that most consistently exhibit decreased functional connectivity (FC) in aMCI patients [22, 31]. Furthermore, some studies have demonstrated that impaired function and compensation coexist within the DMN in aMCI patients [32, 33] and that the altered connectivity of the DMN is linked to the conversion from aMCI to AD [34, 35]. However, the abovementioned studies have primarily focused on a single aspect of the DMN and used independent component analyses or cross-correlation approaches. This is important because information processing within the EM system is thought to involve dynamic interactions among several large-scale neural networks [36].

It is also well established that the prefrontal and parietal regions are crucial aspects of the EF system [37–41]. Using probabilistic independent component analyses or seed-based correlation analyses, several fMRI studies have demonstrated that the fronto-parietal EF network includes the dorsolateral PFC (DLPFC), anterior cingulate cortex (ACC), supplementary motor area (SMA), and orbitofrontal cortex (OFC) [42, 43]. The DLPFC is considered a core region involved in a variety of cognitive tasks, including EF and EM [41], and several studies have verified that EF networks are altered in patients with aMCI and mild AD [44–47]. Several studies identified increased FC between the DLPFC and other regions in aMCI patients [46, 48], whereas others observed disconnections among the regions of the fronto-parietal EF network [43, 49]. These discrepant findings may be related to the different stages of aMCI [50]. Additionally, dynamic functional interactions are thought to play a critical role in the maintenance of daily behavioral function, and the destruction of certain aspects of this connectivity network would likely lead to cognitive decline or disease [51–54]. Therefore, there is a great need to investigate the mechanisms underlying the interaction between the EF and EM networks in aMCI patients.

Regional Homogeneity (ReHo) is a method which can rapidly map the level of regional activity across the whole brain of an individual [55]. Findings from our research group and other studies suggest that brain regions with altered ReHo in patients with aMCI are located in structures associated with the EM [3, 19, 41, 56] and EF [4, 6, 37, 57] networks and include regions such as the hippocampus, PCC/precuneus (PCu), right inferior parietal lobule (IPL), DLPFC, and ventromedial prefrontal cortex (VMPFC) [58–61]. Therefore, the present study computed ReHo values to identify regions with abnormal local connectivity in a group of aMCI patients relative to healthy controls (HC). Next, the crucial overlapping regions (the right RSC and right DLPFC) of the ReHo areas were employed as the seed regions of interest (ROIs) to construct intrinsic EM and EF networks, respectively.

The primary goal of the present study was to investigate differences in resting-state FC patterns in the DLPFC and RSC networks between aMCI patients and HC. It was hypothesized that altered FC might be observed within both of these networks and that impaired function and compensation might coexist in the DLPFC network in patients with aMCI. The secondary goal of the present study was to investigate the mechanisms underlying the interaction between the EF and EM networks in aMCI patients; therefore an alternative hypothesis was that the EF network mediates the processing of EM in this population.

## MATERIALS AND METHODS

### Subjects

The present study included 198 elderly Han Chinese subjects (79 aMCI patients and 119 HC) who were right-handed, between 54 and 80 years of age, had an education level above junior middle school, were in general good health, and had adequate visual and auditory acuity that would allow for successful cognitive testing. The subjects were recruited through media advertisements and community health screening events. The study protocol was approved by the Research Ethics Committee of Affiliated ZhongDa Hospital at Southeast University, and written informed consent was obtained from all subjects prior to participation in the study.

### Inclusion and exclusion criteria

All aMCI patients (including those with single and multiple domains) were diagnosed based on the recommendations of Petersen et al. [62] and others [63] using the following criteria: 1) subjective memory impairment corroborated by the subject and/or an informant; 2) objective memory performance based on a score within ≤1 standard deviation (SD) of age-adjusted and education-adjusted norms on the Auditory Verbal Learning Test (AVLT) 20-minute delayed recall (DR; AVLT-20-DR); 3) normal general cognitive functioning based on a score ≥24 on the Mini Mental State Examination (MMSE) and a score >120 on the Mattis Dementia Rating Scale 2 (MDRS-2); 4) no or minimal impairment in activities of daily living; and 5) absence of dementia or a level not sufficient to meet the criteria of the Diagnostic and Statistical Manual of Mental Disorders, 4^th^ edition, text revision (DSM-IV-TR) for AD. All HC were required to have a normal neurological examination and no complaints of cognitive impairment based on MMSE scores ≥26, MDRS-2 scores >120, and scores on a neuropsychological battery within the normal range. The exclusion criteria were as follows: 1) current existence or a history of cerebrovascular or psychiatric diseases (Hachinski score >4, Hamilton Rating Scale for Depression [HAMD] score >7); 2) gross structural abnormalities revealed by MRI scans; and/or (3) ferrous or electronic implants.

### Clinical evaluation

All subjects underwent a clinical interview performed by trained neuropsychologists (Drs. Shu and Wang) that included a demographic inventory, medical history, and neurological and mental status examinations. General cognitive functioning was evaluated using the MMSE and MDRS-2. Additionally, a neuropsychological battery consisting of the AVLT-20-DR, Rey-Osterrieth Complex Figure Test 20-minute DR (CFT-20-DR), Logical Memory Test 20-minute DR (LMT-20-DR), Stroop Color and Word Tests A, B, and C, and Trail Making Test-A and -B (TMT-A and TMT-B) to evaluate EM and EF functioning was conducted with each subject.

### MRI data acquisition

All MRI scans were obtained at the Affiliated ZhongDa Hospital at Southeast University using a whole-body Siemens Verio 3.0-T scanner (Siemens, Erlangen; Germany) with a standard transmit-receive head coil. All subjects were instructed to relax and close their eyes during the acquisition of the resting-state MRI scans. The resting-state functional images included 240 volumes and were obtained with a gradient-recalled echo-planar imaging (GRE-EPI) sequence using the following parameters: repetition time (TR) = 2000 ms, echo time (TE) = 25 ms, flip angle (FA) = 90°, number of slices = 36, thickness = 4.0 mm, gap = 0 mm, acquisition matrix = 64 × 64, and field of view (FOV) = 240 × 240 mm. High-resolution T1-weighted anatomical images covering the whole brain were acquired by a 3D-magnetization prepared rapid gradient echo sequence with the following parameters: TR = 1900 ms, TE = 2.48 ms, FA = 9°, number of slices = 176, thickness = 1.0 mm, gap = 0 mm, acquisition matrix = 256 × 256, and FOV = 250 × 250 mm.

### Image preprocessing

The raw fMRI data were preprocessed using DPARSF V2.0 Basic Edition (www.restfmri.net/forum/DPARSF) [64] based on the SPM8 toolkit (http://www.fil.ion.ucl.ac.uk/spm) and MATLAB (The MathWorks, Inc.; Natick, MA, USA) programs. The first 10 volumes of data from each subject were discarded to allow for T1 equilibration, and corrections for within-scan acquisition time differences between slices and head motions were made; no participant performed a head motion >2.0 mm of displacement or >2.0° of rotation throughout the course of the scan [65, 66]. Next, the T1 images were coregistered to the mean functional image using a linear transformation, the coregistered T1 images were segmented into gray matter (GM), white matter (WM), and cerebrospinal fluid (CSF), and the head motion-corrected functional images were normalized to a standard template using the transformation matrix estimated from the T1 segmentation [67]. Next, the images were resliced to 3-mm isotropic resolution and subjected to linear detrending and temporal band-pass filtering (0.01-0.08 Hz), and the nuisance signals, including the six head motion profiles and global mean [68, 69], CSF, and WM signals, were regressed out. No significant differences in head motion were observed between the two groups (*p* > 0.05) [65].

### Quality assurance

*GM loss effects:* Numerous studies have observed a significant level of GM atrophy in patients with aMCI [70, 71], and in the present study, the observed differences in FC may have been driven by anatomical differences between the groups. To clarify this issue, a general linear model (GLM) analysis examining the between-group differences in FC was performed using GM volume as an additional covariate [72, 73]. First, the individual GM volume maps were obtained and normalized to the Montreal Neurological Institute (MNI) space using the toolbox of voxel-based morphometry 8 (VBM8; http://dbm.neuro.uni-jena.de/vbm/). Second, the normalized GM volume maps were resampled to the same voxel size as the functional data and further subjected to a logit transformation [logit(a) = 0.5ln(a/1-a)] to improve normality. Third, the voxel-wise values were smoothed with an 8 mm full-width at half-maximum (FWHM) kernel for final statistical analyses. and finally, the resulting GM values were regressed out in a voxel-wise manner as the nuisance regressor from the FC values to control for the influence of GM volume on FC strength. A voxel-wise GM volume correction was performed for each subject, and two-sampletests controlled for age, gender, and years of education were conducted to determine whether there was GM atrophy in the aMCI patients.

#### Head motion effects

Recent resting-state fMRI studies have reported a significant influence of head motion on resting-state FC analyses [65, 66, 74]. To minimize the effects of head motion on the present results, two methods were employed in the quality assurance (QA) measures. First, the head motion effects, which were calculated as the root mean squared (RMS) head displacement and rotation values derived from the motion-correction procedure (in mm and degrees, respectively) were regressed out [68]. Second, a scrubbing procedure was performed on the preprocessed images, then the resting-state FC analyses were performed, and two-independent samples t-tests were conducted to compare between-group differences in head motion parameters between the two groups [13, 65, 66]. Briefly, the framewise RMS deviation (dRMS) values [75] between the neighboring functional volumes within each subject were calculated, and the volumes with a dRMS values >0.5 mm and their adjacent volumes (one back and two forward) were scrubbed for each subject. This procedure partly reduced the bias in the resting-state fMRI signal induced by head motion artifacts [65].

### ReHo calculation

One popular method currently used to analyze RSFC data is a seed correlation analysis in which the seed ROIs are typically selected based on prior anatomical information or previously performed activation maps. However, these types of investigator-dependent selections may not be optimal for evaluating RSFC data because the biases that result from external influences may cause connectivity patterns to exhibit completely different features. In the present study, a novel method was proposed in which the desired seed ROIs were defined in accordance with the nature of the resting-state fMRI data [76].

The approach used in the present study was based on the measurement of ReHo values in the targeted brain areas because the ReHo may more accurately represent the characteristics of brain regions involved in various kinds of activity, as previously described by Zang et al. [77]. Individual ReHo maps were calculated using Kendall's coefficient of concordance (KCC) based on the nearest 27 neighboring voxels across the whole brain because the ReHo could reflect the temporal homogeneity of spontaneous regional activity. First, the ReHo maps were determined within the entire resting brain, and then each ReHo map was divided by the mean ReHo of the whole brain to reduce the effects of individual variability. Then, a smoothing procedure was conducted on the ReHo maps with an 8mm FWHM Gaussian filter to decrease spatial noise.

One-sample t-tests were performed on the individual ReHo maps for each group to establish the intra-group voxel-wise ReHo maps. Then, two-sample t-tests were performed on the individual ReHo maps of the two groups to identify the between-group ReHo differences using voxel-wise GM volumes, age, gender and education as covariates. All data processing was performed with the REST software (Resting-state fMRI Data Analysis Toolkit; (http://resting-fmri.sourceforge.net) [78].

Based on these ReHo findings, four regions with abnormal local connectivity were identified in the aMCI group: the right DLPFC, RSC, superior parietal lobule (SPL), and left parahippocampal gyrus (Figure S1 and Table S1). The right DLPFC and right RSC were selected as seeds for next seed-based RSFC analysis because these two brain regions were the core aspects of the EF and EM networks, respectively[20, 41].

### Seed-based FC analysis

After the calculation of the ReHo values, they were spatially smoothed using an 8-mm FWHM Gaussian kernel on the preprocessed fMRI data. The individual time courses were extracted based on the seed region of the DLPFC, which had been defined as a mask file. For each subject, a mean time series for the ROI was computed as a reference time course, and voxel-wise cross-correlation values between the seed regions and the whole brain were calculated. Then, Fisher's z-transformation was applied to improve the normality of the cross-correlation values [79–81]. The RSC network construction was also completed using the above processes.

### Statistical analysis

#### Demographic and neuropsychological data

All statistical analyses were conducted with SPSS 17.0 software (SPSS, Inc.; Chicago, IL, USA). Two-sample *t*-tests and Chi-square (χ^2^) tests (only utilized for enumeration data) were conducted to compare the demographic data and neuropsychological performances between the two groups. A *p*-value <0.05 was considered to indicate statistical significance.

Composite scores were used in the present study to increase statistical power via reductions in random variability and removing the floor and ceiling effects. First, the raw scores from each test for each subject were transformed into z-scores with reference to the overall means and SD of all subjects. Second, the composite scores were calculated by averaging the z-scores of the individual tests as follows: the EM score included the AVLT-20- DR, CFT-20-DR, and LMT-20-DR scores, and the EF score included the TMT-A, TMT-B, and the Stroop Color and Word C (SCWT-C) tests (*p* < 0.05, Bonferroni-corrected).

### Group-level intrinsic FC analysis

The individual DLPFC and RSC maps for each group were submitted to a random-effect analysis using one-sample *t*-tests with a stringent threshold of *p* < 0.01 and a family-wise error (FWE) correction to reveal the regions that were the most robustly correlated with each seed. Only clusters within the GM mask were retained. Additionally, to avoid ambiguous biological interpretations related to apparently negative connectivity resulting from corrections for global signal changes [69], only positive FC was assessed in the present study.

To evaluate the between-group differences within the DLPFC and RSC networks, a GLM analysis with FC as the dependent variable, group as the independent variable, and age, gender, years of education, and GM volume as covariates was conducted. A statistical threshold of *p* < 0.005 (uncorrected) and a cluster size >1,998 mm^3^ were used to achieve a corrected statistical significance of *p* < 0.01, as determined by a Monte-Carlo simulation (see program AlphaSim by D. Ward).

### Correlation of behavioral performance scores with the intrinsic FC networks

To investigate the neural bases underlying the EF and EM functions of the DLPFC and RSC networks, respectively, a multiple linear regression model analysis was performed. The relationships between the EF and EM scores and the two networks were examined in aMCI patients to determine the behavioral significance of the collaboration of the neural networks (*p* < 0.05, corrected with AlphaSim, cluster size >10,503 mm^3^). Furthermore, to assess whether there is a linear relation between the independent (the EF and EM) and the dependent variable (the DLPFC and RSC networks), we extracted the averaged FC strengths of these regions showing positive and negative correlations between the neuropsychological scores and FC of the DLPFC and RSC network regulates and performed a supplementary correlation analysis (partial correlation) to examine the relationships between the extracted FC strengths and the neuropsychological performance. Then, a conjunction analysis was performed to identify any overlapping regions that were commonly connected with the DLPFC and RSC networks.

### Mediation analysis

Because the present study observed a significant effect of EF on EM performance and the EM deficits in aMCI patients were associated with the right DLPFC functional network (Figure 4), a mediation analysis was conducted to examine whether FC in the right DLPFC network mediated the EF effect on EM performance in aMCI patients. A classic approach was chosen to establish the mediation analysis; a three-step regression model was constructed as follows:

(1) Y = cX + e1
(2) M = aX + e2
(3) Y = c'X + bM + e3,

where X is the dependent variable (EF scores), Y is the independent variable (EM scores), M is the mediator (FC in the DLPFC), a is the regression coefficient for the relationship between EF scores and FC strength, b is the regression coefficient for the relationship between FC strength and EM scores, c is the regression coefficient for the relationship between EF scores and EM scores, and c' represents the effect of EF scores on EM scores while controlling for the indirect effect.

In this analysis, the four conditions used to establish mediation were as follows: 1) c must be significant; 2) a and b were significant; and 3) c' < c (in absolute value, partial mediation) or c' was insignificant (full mediation). Additionally, an indirect ratio was used to present the strength of mediation: ([a*b]/c).

## RESULTS

### Subject characterization

**Figure 1:**
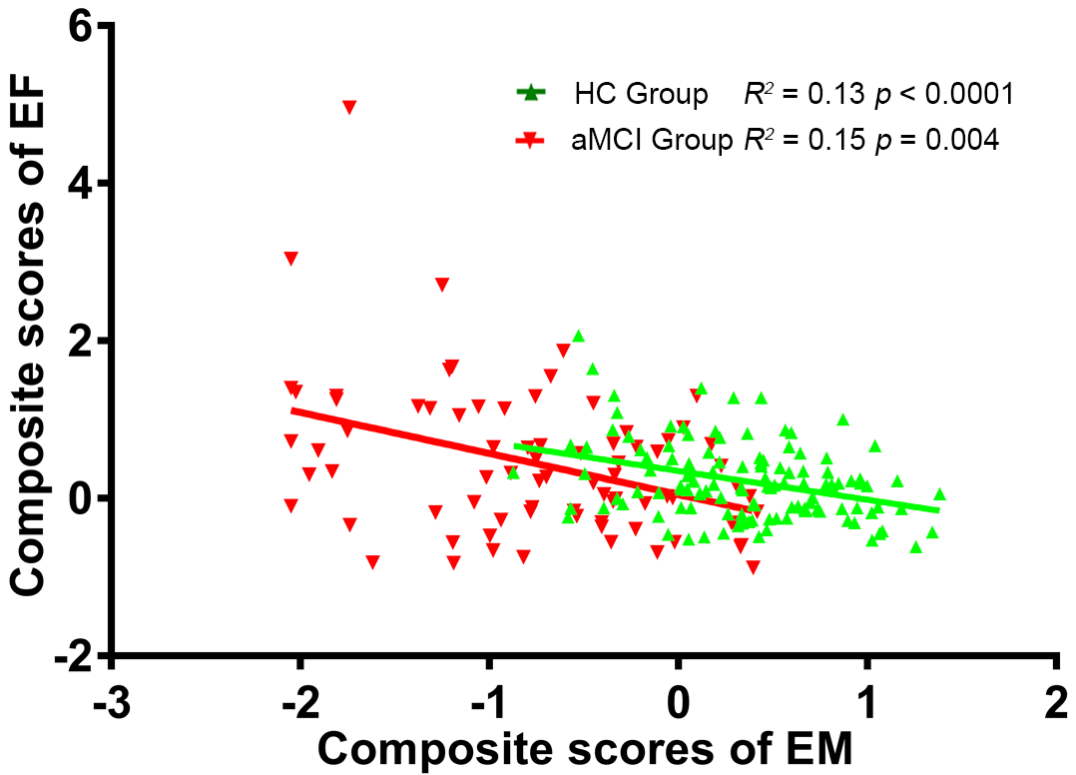
Negative correlation between EM scores and time taken to complete the EF test in the aMCI (R2 = 0.15, *p* = 0.004) and HC (R2 = 0.13, *p* < 0.0001) groups The linear shift to the left indicates decreased EM and EF performance in the aMCI group. Abbreviations: EM, episodic memory; EF, executive function; aMCI, amnestic mild cognitive impairment; HC, healthy controls.

The demographic characteristics of the study subjects are provided in Table 1. No significant differences were observed between the aMCI and HC groups in terms of gender, age, or education level (*p* > 0.05). However, the aMCI group had significantly lower MMSE, EM, and EF scores than the HC group (*p* < 0.01). Additionally, the EM scores were significantly negatively correlated with EF scores in both the aMCI (*R^2^* = 0.15, *p* = 0.0004) and HC (*R^2^* = 0.13, *p* < 0.0001) groups (Figure 1).

**Table 1:** Demographic and neuropsychological data

Characteristics	aMCI(n=79)	HC(n=119)	*p* value
Age(years)	68.16±S6.67	69.65±S7.60	0.148^a^
Gender(male/female)	57/62	42/37	0.562^b^
Education Level(years)	12.43±S3.01	11.84±S3.21	0.188^a^
HAMD	1.87±S3.20	1.71±S2.44	0.681^a^
HIS	1.83±S0.89	1.97±S1.07	0.350^a^
MMSE	26.21±S2.69	28.21±S1.46	0.000^a^
Episodic memory	−0.74±0.71	0.50 0.51	0.000^a^
AVLT-20-min DR	−1.00±0.51	0.65±0.62	0.000^a^
CFT-20-min DR	0.55±1.07	0.42±0.77	0.000^a^
LMT-20-min DR	−0.66±1.00	0.44±0.73	0.000^a^
Executive function	0.44±0.95	−0.27±0.52	0.000^a^
SCWT-C	0.45±1.13	0.28±0.80	0.000^a^
Trail Making Test—A	0.37±1.31	−0.22±0.69	0.000^a^
Trail Making Test—B	0.49±1.28	−0.33±0.64	0.000^a^

Note: Data are presented as mean±SD unless otherwise indicated. Abbreviations: aMCI, amnesic mild cognitive impairment; HC, health control, MMSE, Mini-Mental State Examination; AVLT-20-min DR, Auditory Verbal Learning Test-20-min delayed recall; CFT-20-min DR, Rey–Osterrieth Complex Figure Test with its 20-min delayed recall; LMT-20-min DR, Logical Memory Test-20-min delayed recall; SCWT-C, Stroop Color and Word C; HAMD, Hamilton Depression Scale; HIS, Hachinski Ischemic Score.

a the *p* value was obtained by two-sample two-tailed t test

b the *p* value was obtained by two-tailed χ2 test

### Group-level intrinsic FC

**Figure 2:**
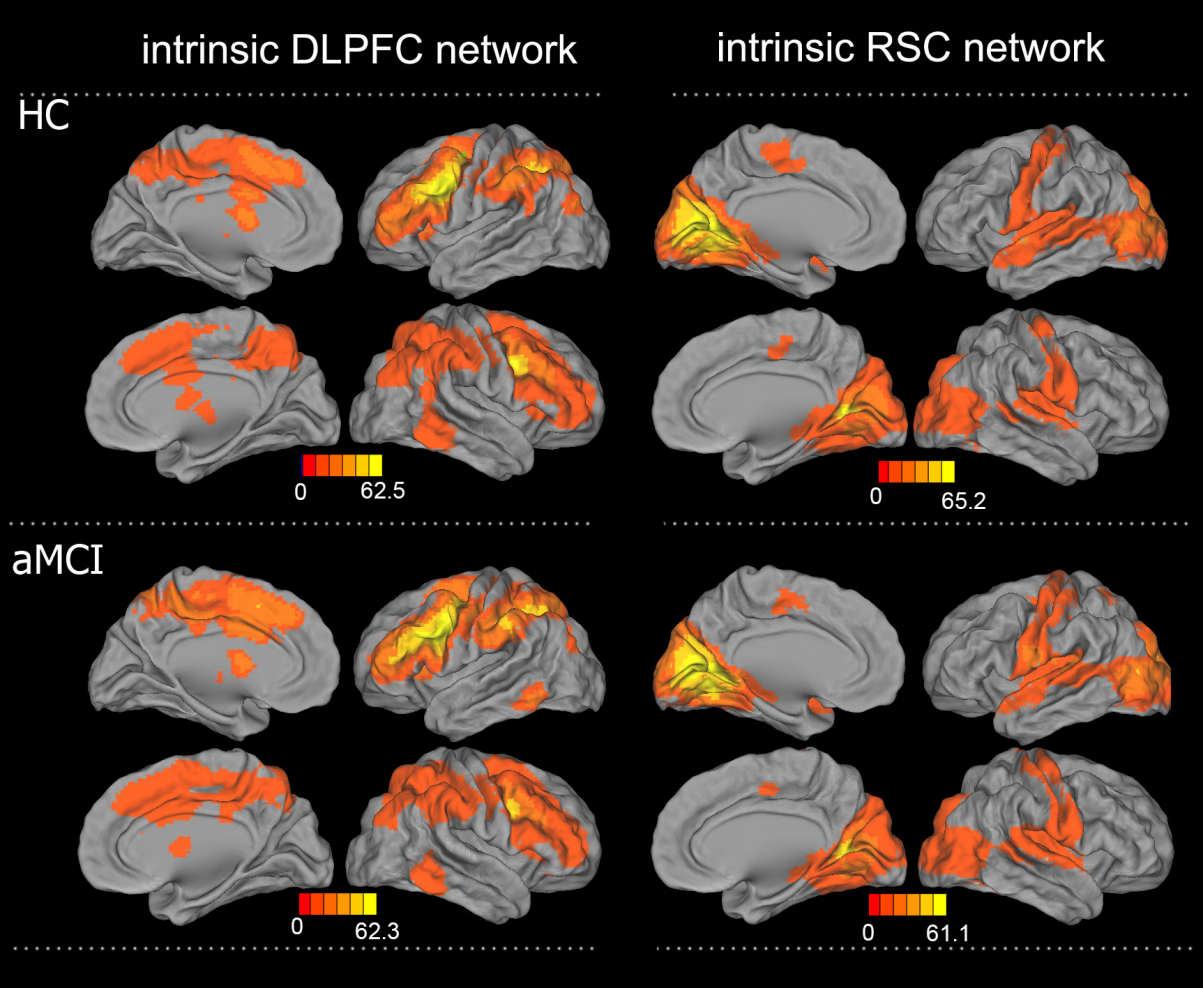
Patterns of intrinsic functional connectivity in the DLPFC and RSC networks in the aMCI and HC groups **A.** DLPFC connectivity network, B: RSC connectivity network (one sample t-test, corrected with AlphaSim, p < 0.01, cluster size > 1,998 mm^3^); color bar presented with z scores. Abbreviations: aMCI, amnestic mild cognitive impairment; HC, healthy controls; DLPFC, dorsolateral prefrontal cortex; RSC, retrosplenial cortex.

**Figure 3:**
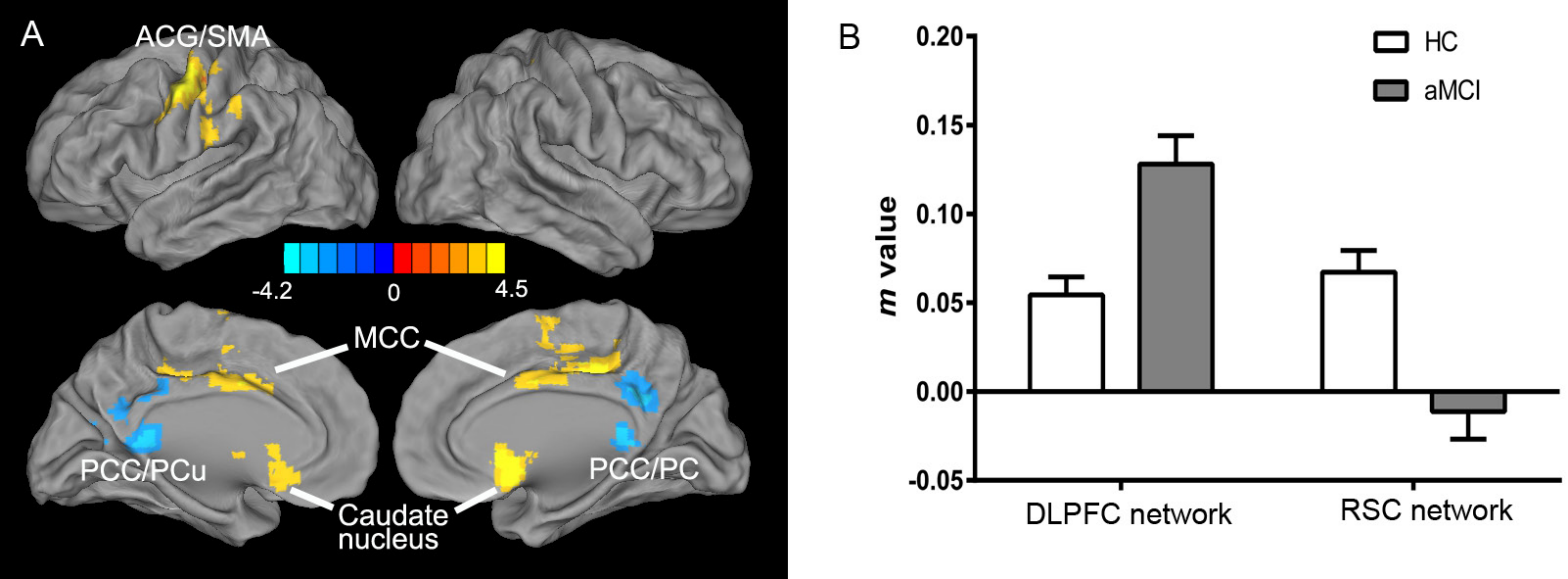
Differential intrinsic functional connectivities in the DLPFC and RSC networks in the aMCI group compared to the HC group (two-sample *t*-test, *p* < 0.05, corrected with AlphaSim) **A.** Increased DLPFC network including the left ACG/SMA, bilateral MCC, and caudate nucleus; decreased RSC network including the bilateral PCC and PCu. A bright color [see note above] indicates increased connectivity and a blue color indicates decreased connectivity; color bar presented with z-scores. **B.** Imbalance in FC strength between the two networks in the aMCI group compared to the HC group (m-value is the Fisher's Z-transformed CC coefficient, as below). Abbreviations: ACG, anterior central gyrus; SMA, supplementary motor area; MCC, middle cingulate cortex; PCC, posterior cingulate cortex; PCu, precuneus; aMCI, amnestic mild cognitive impairment; HC, healthy controls.

The resting-state intrinsic functional network patterns in the DLPFC in both the HC and aMCI groups primarily included the bilateral prefrontal lobe, ACC, MCC, anterior and posterior central gyri, caudate nucleus, and middle temporal gyrus (MTG), and the RSC network patterns included the bilateral hippocampus, parahippocampal gyrus, PCC, posterior central gyrus (PCG), and occipital lobe (Figure 2). Compared to the HC group, the aMCI group exhibited altered connectivity in the DLPFC and RSC networks (Figure 3). In the DLPFC network, the aMCI group showed increased FC in the left anterior central gyrus (ACG), left SMA, bilateral MCC, and caudate nucleus compared to the HC group. In the RSC network, the aMCI group showed decreased FC in the bilateral PCC and PCu compared to the HC group. Details regarding the size, location, and peak density of each cluster are provided in Table 2.

**Table 2:** Clusters with altered intrinsic functional connectivities in the DLPFC and RSC networks in the aMCI group compared to the HC group

Brain regions	Side	FC Network	BA	Cluster voxels	Peak MNI coordinate			Peak intensity
					x	y	z	
Caudate nucleus	L/R	DLPFC	25	119	9	12	−9	3.80
ACG/SMA	L	DLPFC	4, 6	225	−48	−9	54	3.95
MCC	L/R	DLPFC	24	92	12	−24	45	3.37
PCC	L/R	RSC	23, 29	131	1	−48	8	−2.74
PCu	L/R	RSC	31	120	7	−48	37	−3.13

Note: Abbreviation: MNI, montreal neurological institute; x, y, z, coordinates of peak locations in the MNI space; BA, brodmann’s area; R, right; L, left; CN,; ACG, anterior central gyrus; SMA, supplementary motor area; MCC, middle cingulate cortex; PCC, posterior cingulate cortex; PCu, precuneus.

### Behavioral significance of group-level intrinsic connectivity

**Figure 4:**
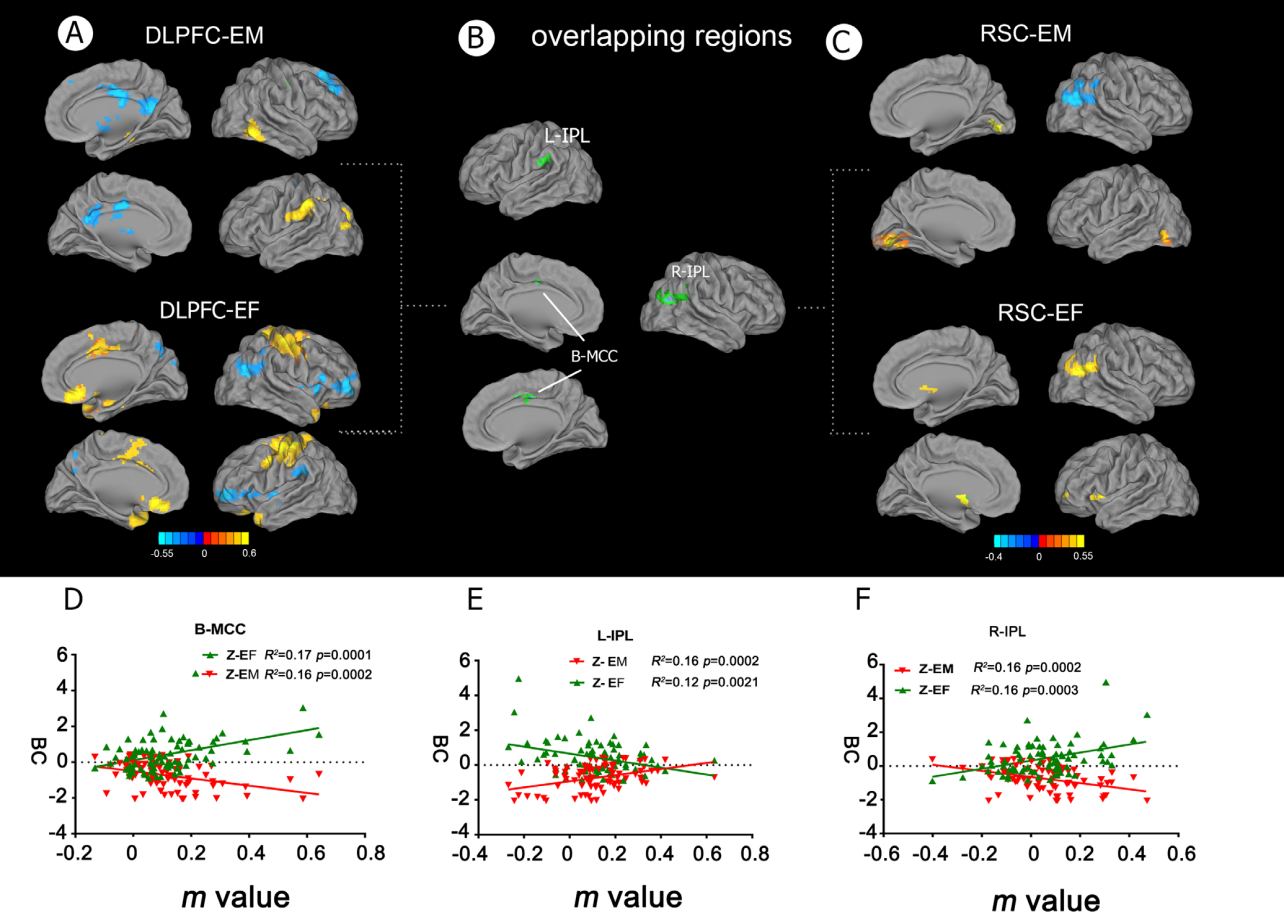
Behavioral significance of altered functional connectivity in the DLPFC and RSC networks in aMCI patients **A.** Neural correlates of the effects of EM and EF in the right DLPFC network. **B.** Overlapping brain regions between impaired EF and memory deficits in the DLPFC and RSC networks, respectively. The interactive regions included the bilateral MCC and IPL. **C.** Neural correlates of the effects of EM and EF in the right RSC network. **D.** and **F.** FC strength in the bilateral MCC and right IPL regions in aMCI patients was negatively correlated with EM and EF capacity (the reverse of time consumed in the EF tests). **E.** FC strength in the left IPL region in aMCI patients was positively correlated with EM and EF capacity (which was the reverse of time consumed in the EF tests). Abbreviations: FC, functional connectivity; IPL, inferior parietal lobule; MCC, middle cingulate cortex; DLPFC, dorsolateral prefrontal cortex; RSC, retrosplenial cortex; EF, executive function; EM, episodic memory; aMCI, amnestic mild cognitive impairment; BC, Behavioral characteristic.

A multivariate linear regression analysis was performed to examine the correlations between DLPFC connectivity and the EF and EM cognitive domains. Positive and negative correlations between the neuropsychological scores and FC of the DLPFC and RSC networks were observed in a number of brain regions (Figure 4A and 4C), and overlapping brain regions were identified in the bilateral MCC and left IPL (Figure 4B and Table 3). In the DLPFC network, the MCC was positively correlated with EF, but negatively correlated with EM (Figure 4D), whereas the left IPL was negatively correlated with EF, but positively correlated with EM (Figure 4E). In the RSC network, an overlapping region was identified in the right IPL (Figure 4B and Table 3), which was positively correlated with EF, but negatively correlated with EM (Figure 4F).

**Table 3:** Neural bases of the interaction between episodic memory function and executive function in the DLPFC and RSC functional connectivity networks

Brain regions	Side	FC Network	BA	Cluster voxels	Peak MNI coordinate			Peak intensity
					x	y	z	
**IPL**	**L**	**DLPFC**	**39**	**122**	**-56**	**-36**	**33**	**-**
**MCC**	**L/R**	**DLPFC**	**24**	**365**	**21**	**0**	**24**	**-**
**IPL**	**R**	**RSC**	**39, 40**	**283**	**45**	**-69**	**21**	**-**

Note: Abbreviation: MNI, montreal neurological institute; x, y, z, coordinates of peak locations in the MNI space; BA, Brodmann’s area; R, right. L, left. IPL, inferior parietal lobule; MCC, middle cingulate cortex;

### Mediation analysis

**Figure 5:**
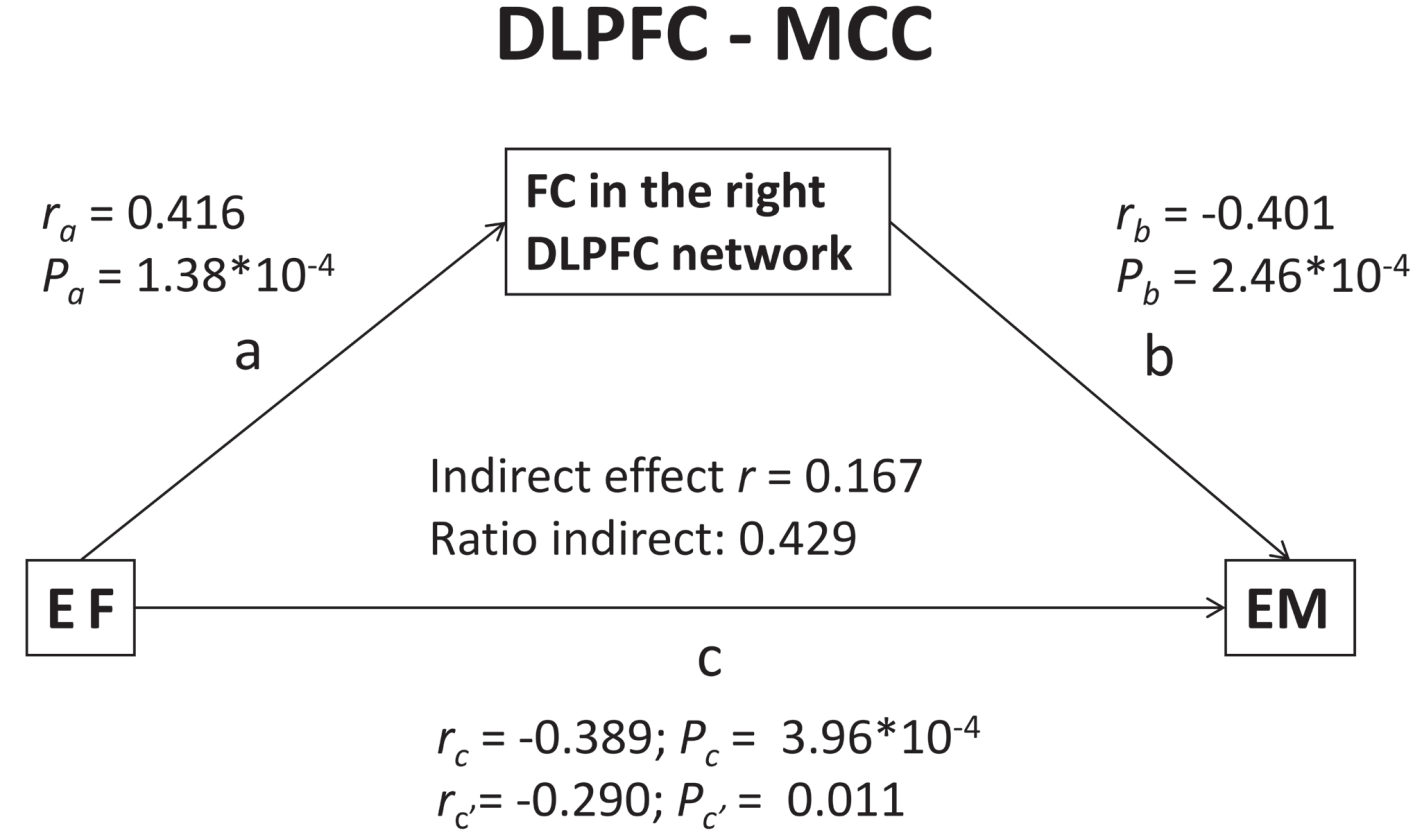
Results of the mediation analysis FC between the DLPFC and the bilateral MCC had an indirect positive effect, representing 42.9% of the total effect on the association between EF and EM performance in aMCI patients. Abbreviations: FC, functional connectivity; MCC, middle cingulate cortex; DLPFC, dorsolateral prefrontal cortex; EF, executive function; EM, episodic memory; aMCI, amnestic mild cognitive impairment.

The mediation analyses revealed that the connectivity between the right DLPFC and bilateral MCC mediated the association between EF and EM in patients with aMCI. These findings indicate that the effects of EF on EM performance in aMCI patients may be explained by two mechanisms: the direct effect of EF on EM or an indirect pathway mediated via the effects of EF on the DLPFC network. More specifically, FC between the DLPFC and bilateral MCC accounted for 42.9% of the indirect positive effect on the association between EF and EM performance in patients with aMCI (Figure 5).

## DISCUSSION

The present study investigated activity in the EF and EM networks to more fully characterize the mechanisms underlying the interaction between memory deficits and executive dysfunction in patients with aMCI. Three main results of the present study should be emphasized. First, the EM scores were significantly negatively correlated with the EF scores in both the HC and aMCI groups. Second, there were increases in the intrinsic EF network and decreases in the EM network, suggesting that the neural activity between the EF and EM networks was imbalanced in the aMCI patients. Third, the connectivity between the right DLPFC and bilateral MCC mediated the association between EF and EM performance in aMCI patients. These findings provide direct evidence supporting the suggestion that EF mediated EM performance at the network level in a non-demented elderly population.

The present study identified disruptions in ReHo in the right DLPFC and RSC, which have consistently been identified as core regions of the EF and EM networks, respectively [20, 39]. Converging evidence from our research group and others indicates that ReHo is altered in the RSC in the EM network [3, 19, 41, 56] and in the DLPFC in the EF network [4, 6, 37, 57] in aMCI patients. Therefore, it was reasonable for the present study to utilize a new approach to analyze FC that employed the ReHo results as the seed ROIs. Furthermore, this new method of FC analysis likely has advantages over other popular protocols that are currently used and may have provided more reasonable and persuasive results.

The neuropsychological results in the present study showed that EF and EM were impaired in aMCI patients and that there was a significant relationship between these two crucial cognitive domains such that EF performance was significantly and positively related to EM. However, EF is more commonly impaired in AD patients than aMCI, several studies did not find a significant difference between aMCI patients and normal controls in terms of these cognitive domains [82, 83]. This may be partially due to complex neurophysiologic mechanisms of the disease and dynamic changes in EF during different stages of aMCI. We confirm that the emergence of EF deficits significantly accelerates the conversion rate from aMCI to AD [9].

In the present study, a decreased RSC FC network and an increased DLPFC FC network were observed in aMCI patients. These findings suggest an imbalance between intrinsic EF connectivity and intrinsic EM connectivity in this population. Several studies using positron emission tomography (PET) have demonstrated that the RSC is one of brain regions that undergoes metabolic decline in patients with AD and aMCI [21, 84, 85]. Similarly, several fMRI studies have found that the PCC and RSC regions have significantly disrupted connectivities in patients with aMCI [86, 87]. Taken together, the abovementioned studies and the present findings suggest that the RSC network is altered and exhibits decreased FC in aMCI patients. However, the nature of activity in the DLPFC network remains elusive based on the findings of previous studies. Some fMRI studies have observed increased FC between the right DLPFC [48, 88] and other regions, whereas other studies have found decreased FC in the fronto-parietal network in aMCI patients [43, 79]. Longitudinal follow-up studies have verified that activity within the fronto-parietal network appears to decrease with the progression of the disease [89]. The present findings may imply that a compensatory mechanism exists to account for a disconnection of the EM network in aMCI patients. Although this population showed increased intrinsic FC in the DLPFC network in the present study, this was likely a pathogenic mechanism that is reflective of an unsuccessful attempt to recruit preserved neuronal areas as a compensation for pathology [90, 91].

The present study further elucidated the neural bases of the collaboration between EM and EF in aMCI patients by revealing that the main overlapping regions underlying EF and EM in the right DLPFC and RSC networks, respectively, were the bilateral MCC and the IPL. It is well documented that the IPL is a heterogeneous area involved in multimodal functions such as sensory motor processing [92], executive control [93], salience detection [42], and EM function [19]. The present findings may be explained by the fact that the parietal cortex and the cingulate cortex play crucial roles in the processing of EF and EM functions [3, 43].

Interestingly, the present study also observed that EF had a significant effect on EM and that the EM deficits in aMCI patients were associated with the right DLPFC functional network. Our study verified that FC in the right DLPFC network mediated the effects of EF on EM performance in aMCI patients. The connectivity between the DLPFC network and the bilateral MCC had an indirect and significant positive effect on the association between EF and EM performance in aMCI patients. Evidence from neuroimaging studies indicates that the MCC is reciprocally connected with fronto-parietal regions, particularly the DLPFC [94]. Taken together, the present findings are the first to verify that the MCC is a core region of the EF network that might mediate the processing of EM.

It should be noted that there are several limitations to the present study. First, the recruitment of the aMCI patients was based on clinical criteria, and patients with partial symptomologies, such as prodromal AD according to the new AD diagnostic criteria [95], may therefore have been included in the study. In future studies, a lumbar puncture should be performed to distinguish patients with prodromal AD or MCI from those with AD [2, 96]. Second, the present study was a cross-sectional investigation. The observed changes in the patterns of network connectivity may have reflected a dynamic phenomenon, and these changes may have appeared to be greater than they actually were; thus, the described deficits in FC may have reflected the state of the disease rather than to particular subtypes. Thus, additional longitudinal studies will be very helpful in determining whether the changes in the patterns of network FC in aMCI patients are specifically associated with a more rapid course of AD. Third, the RSC was selected as the seed in the FC analyses because it is a key node in the retrieval of EM [20], but this area is not a classic brain region associated with EM encoding, consolidation, or retrieval processing [3, 19]. Further studies are required to apply the approaches used in the present study to investigate the memory network at the whole-brain level

In summary, the present findings revealed a simultaneous disconnection of the intrinsic RSC network and compensation for this within the DLPFC network in aMCI patients. Furthermore, these abnormal network activities were associated with impairments of EF and EM. The present findings provide new insights into the neural mechanisms underlying the interaction between impaired EF and memory deficits in patients with aMCI, and suggest that the EF network may mediate EM performance in this population.

## SUPPLEMENTARY MATERIAL FIGURE AND TABLE


