# Supplementary material for: Mediation of episodic memory performance by the executive function network in patients with amnestic mild cognitive impairment: a resting-state functional MRI study
Source: Oncotarget. 2016 Aug 31;7(40):64711–25. doi: 10.18632/oncotarget.11775 (PMC5323110; doi:10.18632/oncotarget.11775)
Supplement: Supplementary file 1 [file oncotarget-07-64711-s001.pdf]

Mediation of episodic memory performance by the executive function network in patients with amnestic mild cognitive impairment: a resting-state functional MRI study

Supplementary Material

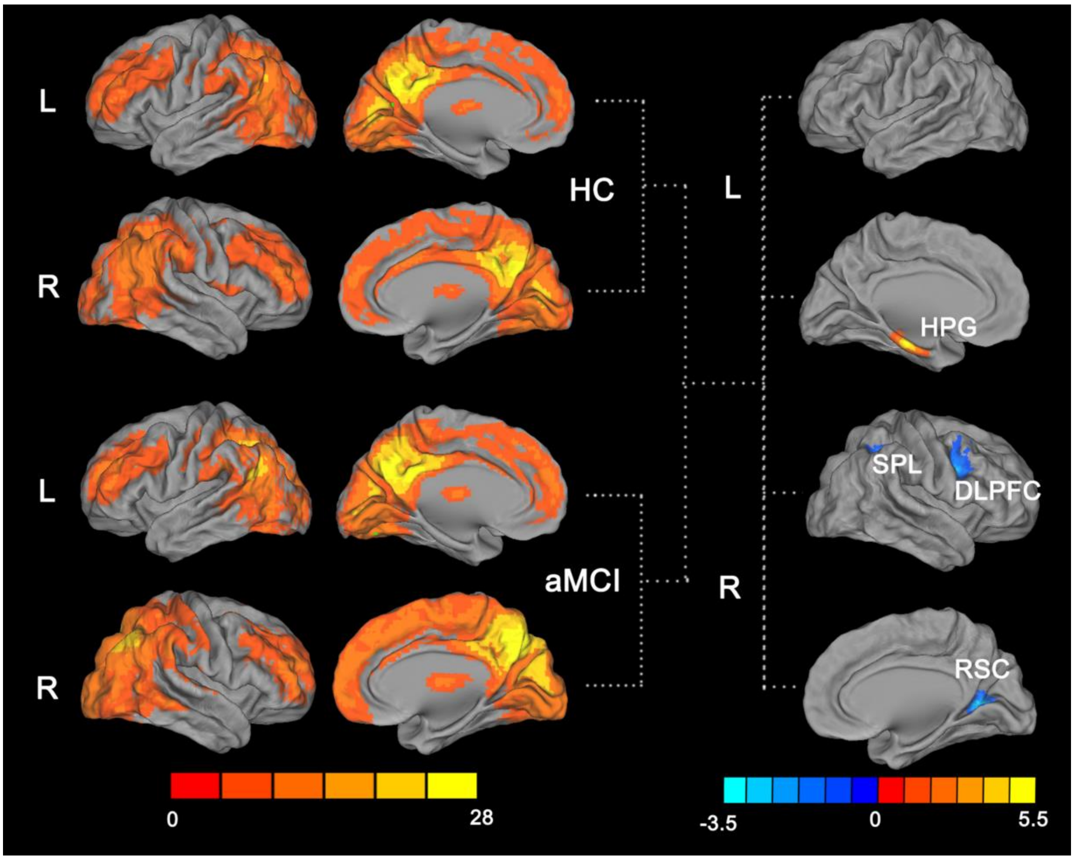

Figure S1. Patterns and group differences in ReHo. Left: ReHo patterns in the HC and aMCI groups; Right: Brain regions in the aMCI group with significantly altered ReHo compared to the HC group. A bright color indicates increased ReHo, and a blue color indicates decreased ReHo; Results projected on a surface template (Caret software; Van Essen, 2005). Abbreviations: ReHo, regional homogeneity; aMCI, amnesic mild cognitive impairment; HC, healthy controls; HPG, parahippocampal gyrus; SPL, DLPFC, dorsolateral prefrontal cortex; SPL, superior parietal lobule; RSC, retrosplenial cortex.

Table S1. Brain regions with altered ReHo in the aMCI group compared to the HC group.

Brain regions	Side	BA	Cluster voxels	Peak MNI coordinate			Peak intensity
				x	y	z	
DLPFC	R	9	110	38	11	34	-2.97
SPL	R	7	69	36	-42	42	-3.34
RSC	R	30	64	21	-63	8	-2.80
PHG	L	36	32	-21	-21	-23	2.70

Notes: Abbreviation: BA, brodmann's area; MNI, montreal neurological institute; x, y, z, coordinates of peak locations in the MNI space; R, right; L, left; DLPFC, dorsolateral prefrontal cortex; SPL, superior parietal lobule; RSC, retrosplenial cortex; PHG, parahippocampal gyrus.
